# Monitoring of *KRAS*-mutated ctDNA to discriminate pseudo-progression from true progression during anti-PD-1 treatment of lung adenocarcinoma

**DOI:** 10.18632/oncotarget.16935

**Published:** 2017-04-07

**Authors:** Nicolas Guibert, Julien Mazieres, Myriam Delaunay, Anne Casanova, Magali Farella, Laura Keller, Gilles Favre, Anne Pradines

**Affiliations:** ^1^ Thoracic Oncology Department, Larrey Hospital, University Hospital of Toulouse, Toulouse, France; ^2^ Inserm, Centre de Recherche en Cancérologie de Toulouse, CRCT UMR-1037, Toulouse, France; ^3^ Institut Claudius Regaud, IUCT-Oncopole, Laboratoire de Biologie Médicale Oncologique, Toulouse, France; ^4^ University of Toulouse III (Paul Sabatier), Toulouse, France

**Keywords:** immunotherapy, anti-PD-1, non-small-cell lung cancer, KRAS mutation, circulating tumor DNA

## Abstract

**Objectives:**

Pseudo-progression is a rare but worrying situation for both clinicians and patients during immunotherapy. Dedicated ir-RECIST criteria have been established to improve this situation. However, this can be sometimes considered inadequate and patients experiencing true progression may then receive inefficient treatments. Additional reliable tools to discriminate pseudo from true progression are thus needed. So far, no biomarker has been identified to distinguish pseudo from true progression. We hypothesize that biomarkers associated with the molecular characteristics of the tumor may be of interest. To avoid a tumor re-biopsy, circulating markers appear to be a less invasive and reproducible procedure. As ctDNA kinetics correlate with the response to treatment in *KRAS*-mutated adenocarcinoma, we anticipated that this analysis could be of interest.

**Materials and methods:**

We monitored the level of *KRAS*-mutated ctDNA by digital droplet PCR in serial plasma samples from two patients who had experienced pseudo-progression and compared the variations with those from of a patient that had true progression.

**Results:**

ctDNA showed rapid and dramatic decreases in pseudo-progressive patients, whereas it was strongly increased in the progressive patient.

**Conclusions:**

ddPCR of ctDNA may thus be an additional tool to discriminate pseudo-progression from true progression for tumors that harbor an oncogenic addiction.

## INTRODUCTION

Immunotherapy that targets the PD-1/PD-L1 checkpoint has become an appealing advance to treat NSCLC since the development of targeted therapies. However, major pitfalls that preclude the use of these new agents are still challenging: i.e., *i)* a lack of strong biomarkers to make a reliable selection of the best candidate patients; this is because tissue PD-L1 expression is a good but not unerring predictive factor for a response [1]; and *ii)* the need for new tools to dynamically evaluate responses.

CT evaluations are sometimes undermined in cases of pseudo-progression, despite the development of dedicated immune-related RECIST criteria [2]. Pseudo-progression and immune-related patterns of mixed response are particularly challenging. This event is not rare, affecting 10 to 15% of patients treated by PD-1 inhibitors for metastatic malignant melanoma, and is usually overtaken by ir-RECIST criteria [3, 4]. Nevertheless, even taking into account this new classification, some of these patients have true disease progression and should be rapidly switched to alternative treatments. Additional markers are needed to assess responses to immunotherapy and to help make therapeutic decisions to ensure that an ineffective treatment is discontinued.

We have recently reported on the use of cell-free DNA (cfDNA) to monitor tumor burden during the treatment of *KRAS*-mutated adenocarcinoma [5], a mutation known to be associated with improved outcomes under anti-PD-1 therapy because of its high mutational burden and PD-L1-expression rate [6, 7]. We thus supposed that cfDNA mutation analysis could be an additional tool to be used during the follow-up of this subpopulation of patients.

## CASE REPORTS

Two patients with metastatic *KRAS*-mutated adenocarcinoma, where pseudo-progression was observed during anti-PD-1 treatment, were included in this study. Another, who did not respond to immunotherapy, was used as a control. These three patients had a *KRAS* mutation previously detected in formalin-fixed paraffin-embedded tissue samples and plasma samples had been prospectively collected (clinical trial NCT02827344) at initiation of the PD-1 inhibitor (T0), after two cycles (T1), and then at each radio-clinical evaluation (T2,3,…).

For each patient, two 5-mL blood samples were collected and used to isolate cfDNA using a circulating nucleic-acid kit (Qiagen). cfDNA was then tested for the presence of the corresponding *KRAS* mutation using digital droplet PCR (QX200, Bio-Rad). The input DNA was emulsified into 20,000 droplets, amplified by PCR with specific TaqMan probes, and then analyzed by flow cytometry. Genomic DNA from H23, H441 and A427 cell lines was used as a positive control to detect *KRAS*-G12C, *KRAS* G12V and *KRAS*-G12D mutations, respectively. Specificity of the assay was assessed using samples derived from *KRAS* wild-type patients.

## RESULTS

**Figure 1:**
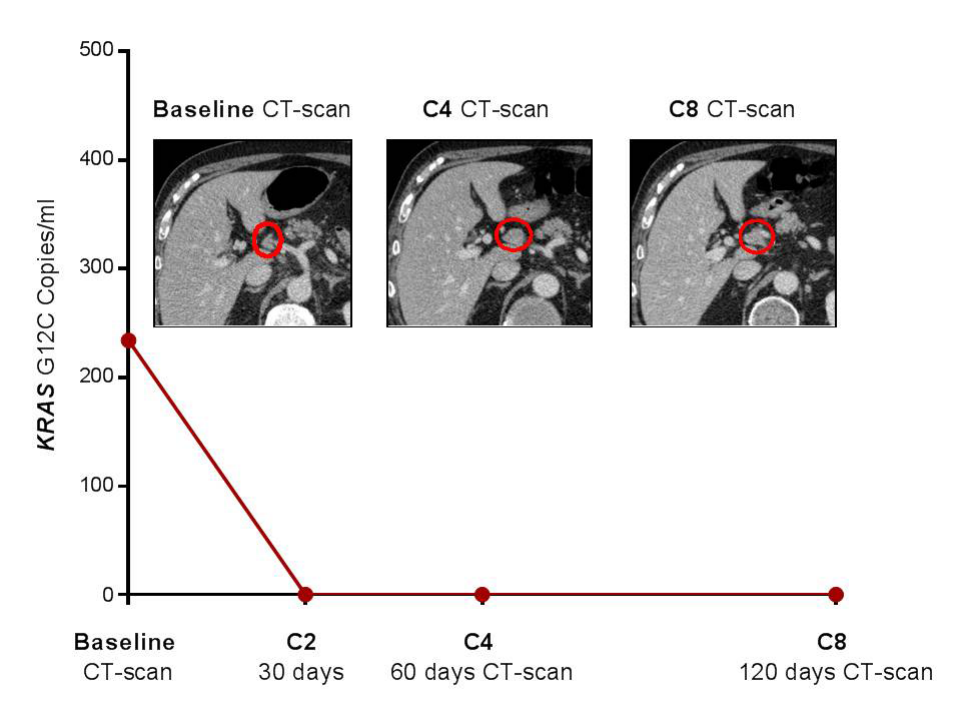
Pseudo-progression of abdominal nodes metastases in patient # 1 after four courses of nivolumab, which was confirmed by favourable outcomes at the second evaluation Concomitant early and complete plasma response after 2 cycles of treatment.

**Figure 2:**
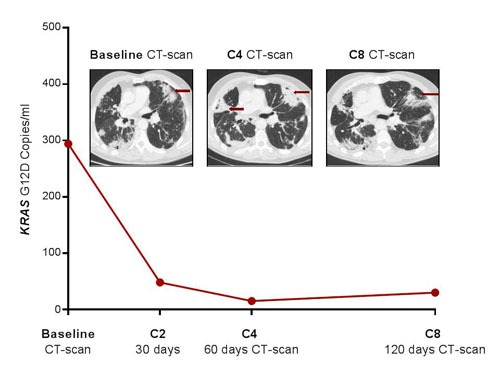
Pseudo-progression of lung metastases in patient # 2 after four courses of nivolumab, which was confirmed by favourable outcomes at the second evaluation Early and dramatic decrease in *KRAS*-mutated ctDNA.

The two patients with pseudo-progression showed high levels of *KRAS*-mutated ctDNA at baseline but an early and dramatic decrease after the first courses of treatment (Table 1 and Figure 1). The first patient (*KRAS*-G12C-mutated adenocarcinoma) showed a mixed response with progression of hilar hepatic-node metastases (28 vs. 15 mm) and apparition of supra-centimetric latero-aortic nodes, but stability of pulmonary lesions, and was considered to have disease progression by both RECIST and irRECIST criteria (Figure 1). Nevertheless, nivolumab was pursued due to clinical benefit considering the eventuality of pseudo-progression. Patient showed a complete plasma response after only two cycles of nivolumab, confirmed at the time of the first CT-scan evaluation, and nivolumab was then maintained for 16 more cycles (Table 1). After four courses of nivolumab, the second patient (a *KRAS*-G12D-mutated adenocarcinoma) also reported a clinical benefit, contrasting with a worrying condensation of lung metastases and a global increase in tumor burden on the first CT-scan (Figure 2). The next CT evaluation showed a partial response and the patient responded to nivolumab for 17 cycles. In contrast, the level of *KRAS*-mutant ctDNA showed an early and dramatic decrease (Table 1). The scanographic and plasma responses are shown on Figure 2.

**Table 1:** Variation in *KRAS*-mutated DNA in plasma during treatment with nivolumab

	Time of blood collection	Last treatment received (before the time of blood collection)	Mutant copies/mL in cfDNA	irRECIST evaluation /RECIST 1.1 evaluation
Patient 1 ***KRAS* G12C**	T0 T1 T2 (1st scan) T3 (2nd scan)	chemotherapy nivolumab nivolumab nivolumab	234 0 0 0	**DP/DP** PR
Patient 2 ***KRAS*** **G12D**	T0 T1 T2 (1st scan) T3 (2nd scan)	chemotherapy nivolumab nivolumab nivolumab	294 48 15 30	**DP/DP** PR
Patient 3 ***KRAS*** **G12V**	T0 T1 T2 (1st scan)	chemotherapy nivolumab nivolumab	1696 32800 19200	**DP**

Abbreviations: cfDNA: circulating free DNA; irRECIST: immune-related Response Evaluation Criteria in Solid Tumors. PD: progressive disease. PP: pseudo-progression. PR: partial response. SD: stable disease.

**Figure 3:**
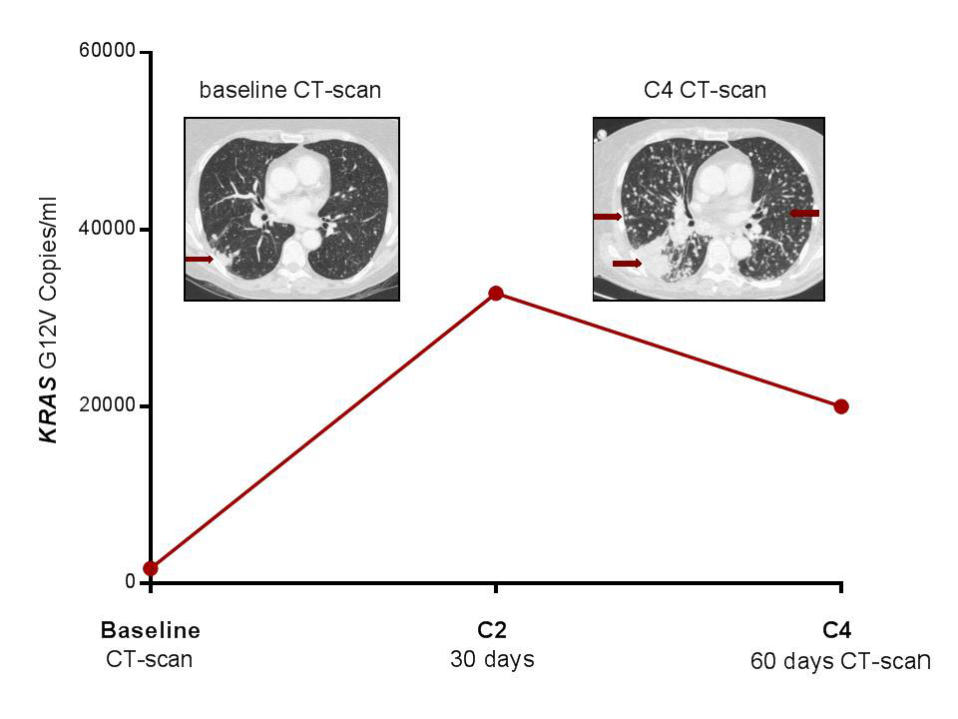
Progression of pulmonary metastases in patient # 3 after four courses of nivolumab Concomitant plasma progression with dramatic increase in *KRAS*-mutated ctDNA.

The third patient that had disease progression (Figure 3) after four cycles of nivolumab had a concordant plasma response (with a 10-fold increase after only 2 cycles) of *KRAS*-G12V-mutated ctDNA (Table 1). This last patient had a higher baseline tumor burden (pulmonary, liver and bone metastases), explaining the higher ctDNA level.

## DISCUSSION

We have recently reported on the usefulness of cfDNA to monitor responses to treatment of *KRAS*-mutated lung adenocarcinoma. Here we have shown that this tool could be used to discriminate pseudo from true progression in patients experiencing increased tumor burden, as seen on the first CT evaluation. Ir-RECIST criteria have been structured to help clinicians make decisions of whether to pursue immunotherapy or not in these patients when imaging information is insufficient to make this decision. Tolerance to treatment, the subjective benefit reported by the patient, and the experience of the clinician are also important. However, additional reliable tools would be very helpful.

The kinetics between mutations in cfDNA levels at baseline and after the first cycles of nivolumab may allow patients with true progression to be rapidly redirected to receive alternative options. Thoracic oncologists are still familiarized with conventional response evaluations that are used with cytotoxic agents.

The two patients reported here would have been considered as having progressive disease according to RECIST 1.1 but also irRECIST criteria. Thus, further information to reinsure clinicians and patients make the appropriate decision regarding treatment would be of great interest. Plasma ddPCR has been shown to be an inexpensive and very reliable test with a short turnaround time of 3 (1-7) days [8].

Our data are obviously too limited to make definitive conclusions and should be validated in larger cohorts; however, this is a challenge as pseudo-progression is a rare event. Another pitfall is the need to identify a molecular alteration to target. The quantification of whole cfDNA (wild type and mutated) cannot be used for this purpose, as its specificity is too low for a reliable follow-up of tumor burden, which is affected by numerous benign situations that increase its level [9]. This drawback is overcome by directly targeting the mutated oncogene in the plasma; thus, our results may be extrapolated to other oncogenic drivers, like *EGFR* of *BRAF*, for which variations in cfDNA have been shown to be concordant with outcomes [10, 11].
